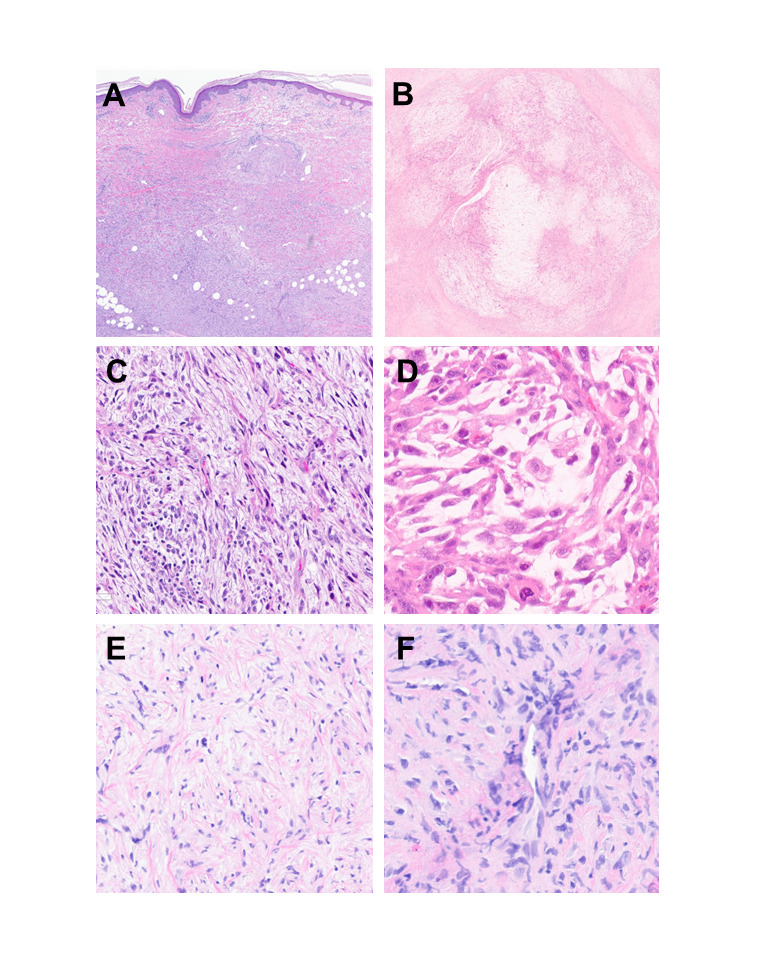# Pan-cancer Genomic Analysis of *AXL* Mutations Reveals a Novel, Recurrent, Functionally Activating *AXL* W451C Alteration Specific to Myxofibrosarcoma

**DOI:** 10.1097/PAS.0000000000002191

**Published:** 2024-02-19

**Authors:** Erik A. Williams, Isabella Vegas, Fardous F. El-Senduny, Jessica Zhang, Douglas A. Mata, Matthew C. Hiemenz, Sarah R. Hughes, Brianna C. Sa, Garrett P. Kraft, Nicole Gorbatov, Kathleen Foley-Peres, Edward Z. Sanchez, Clara Milikowski, Kevin Jon Williams, Jeffrey S. Ross, Razelle Kurzrock, Elizabeth A. Montgomery, David B. Lombard, Surinder Kumar

**Affiliations:** * Department of Pathology and Laboratory Medicine, University of Miami, Sylvester Comprehensive Cancer Center; † Department of Pathology, Jackson Memorial Hospital, Miami, FL; ‡‡ Department of Pathology, Miami VA Healthcare System, Miami, FL; ¶ Department of Radiology, St. Anthony’s Hospital, St. Petersburg, FL; ‡ Foundation Medicine, Inc., Cambridge, MA; ∥ Department of Biology, Bristol Community College, Fall River, MA; § Department of Pathology, Gundersen Health System, La Crosse; †† Department of Medicine, Medical College of Wisconsin, Milwaukee, WI; # Departments of Physiology and Medicine, Lewis Katz School of Medicine, Temple University, Philadelphia, PA; ** Department of Pathology, State University of New York Upstate Medical University, Syracuse, NY

**Keywords:** sarcoma, myxofibrosarcoma, AXL, next-generation sequencing

## Abstract

Myxofibrosarcoma (MFS) is a common soft tissue sarcoma of the elderly that typically shows low tumor mutational burden, with mutations in *TP53* and in genes associated with cell cycle checkpoints (*RB1*, *CDKN2A*). Unfortunately, no alterations or markers specific to MFS have been identified and, as a consequence, there are no effective targeted therapies. The receptor tyrosine kinase AXL, which drives cellular proliferation, is targetable by new antibody-based therapeutics. Expression of *AXL* messenger RNA is elevated in a variety of sarcoma types, with the highest levels reported in MFS, but the pathogenic significance of this finding remains unknown. To assess a role for AXL abnormalities in MFS, we undertook a search for *AXL* genomic alterations in a comprehensive genomic profiling database of 463,546 unique tumors (including 19,879 sarcomas, of which 315 were MFS) interrogated by targeted next-generation DNA and/or RNA sequencing. Notably, the only genomic alterations recurrent in a specific sarcoma subtype were *AXL* W451C (n = 8) and *AXL* W450C (n = 2) mutations. The tumors involved predominantly older adults (age: 44 to 81 [median: 72] y) and histologically showed epithelioid and spindle-shaped cells in a variably myxoid stroma, with 6 cases diagnosed as MFS, 3 as undifferentiated pleomorphic sarcoma (UPS), and 1 as low-grade sarcoma. The *AXL* W451C mutation was not identified in any non-sarcoma malignancy. A review of publicly available data sets revealed a single *AXL* W451C-mutant case of UPS that clustered with MFS/UPS by methylation profiling. Functional studies revealed a novel activation mechanism: the W451C mutation causes abnormal unregulated dimerization of the AXL receptor tyrosine kinase through disulfide bond formation between pairs of mutant proteins expressing ectopic cysteine residues. This dimerization triggers AXL autophosphorylation and activation of downstream ERK signaling. We further report sarcomas of diverse histologic subtypes with *AXL* gene amplifications, with the highest frequency of amplification identified in MFS cases without the W451C mutation. In summary, the activating *AXL* W451C mutation appears highly specific to MFS, with a novel mechanism to drive unregulated signaling. Moreover, *AXL* gene amplifications and messenger RNA overexpression are far more frequent in MFS than in other sarcoma subtypes. We conclude that these aberrations in *AXL* are distinct features of MFS and may aid diagnosis, as well as the selection of available targeted therapies.

Myxofibrosarcoma (MFS) is a common adult soft tissue sarcoma that most frequently occurs in the elderly. The tumor shares overlapping genomic features with undifferentiated pleomorphic sarcoma (UPS), including widespread copy number alterations and often low tumor mutational burden (TMB, mutations/Mb) with mutations in *TP53* and in genes associated with cell cycle checkpoints (*RB1, CDKN2A*).^1–3^ Moreover, methylation array studies have shown that MFS and most cases of UPS cluster similarly, implying that they constitute a morphologic spectrum of a single disease.^4^ Although MFS has distinct histopathologic features, no recurrent genetic alterations specific to MFS/UPS have been identified and, as a consequence, there are no effective targeted therapies.

The tyrosine kinase AXL, which drives cellular proliferation, is a single-pass transmembrane protein and a member of the Tyro3-AXL-MerTK family of receptors. AXL is activated in many cancers, with an important role in cell proliferation, motility, and the promotion of an immunosuppressive local microenvironment. In sarcomas specifically, *AXL* messenger RNA (mRNA) expression levels are significantly elevated in comparison with levels in other cancer types and in normal tissue.^5^ Among sarcomas, *AXL* mRNA expression is highest in MFS.^5^ Accordingly, a patient-derived xenograft study demonstrated that MFS showed the highest and most stable expression of AXL protein compared with other sarcoma types.^6^ Nevertheless, the pathogenic significance of these findings remains unknown. Characterization of *AXL* genomic alterations, either point mutations or copy number alterations (amplifications) in sarcomas, including MFS, has been limited.^4,7^ To assess the role of AXL abnormalities in MFS, we sought to characterize the genomics of *AXL* across sarcoma types. We identified 2 specific *AXL* mutations, as well as an unusually high frequency of *AXL* gene amplifications, as distinct genomic features of MFS.

## METHODS

### Sample Selection and Comprehensive Genomic Profiling

Our archive of 463,546 tumor samples, including 19,879 sarcomas, 315 of which were MFS, each from a different patient, underwent comprehensive genomic profiling (CGP) in a Clinical Laboratory Improvement Amendments–certified, College of American Pathologists–accredited laboratory (Foundation Medicine Inc.). This cross-sectional study included patient samples that had been sent as part of routine care from medical care facilities across North America from January 2014 to March 2022 for identification of potentially targetable genetic alterations. Approval for this study, including a waiver of informed consent and a Health Insurance Portability and Accountability Act waiver of authorization, was obtained from the Western Institutional Review Board (Protocol No. 20152817). The present study was conducted according to the guidelines of the Declaration of Helsinki.

For CGP, sections were macrodissected to achieve >20% estimated percentage of tumor nuclei in each case, where percentage of tumor nuclei = 100 times the number of tumor cells divided by the total number of nucleated cells. Next, ≥60 ng DNA was extracted from 40 μm sections cut from tumor samples in formalin-fixed, paraffin-embedded tissue blocks. The samples were assayed by adaptor ligation hybrid capture, performed for all coding exons of 236 (v1), 315 (v2), or 405 (v3) cancer‐related genes plus select introns from 19 (v1), 28 (v2), or 31 (v3) genes frequently rearranged in cancer (Supplemental Table 1, Supplemental Digital Content 1, http://links.lww.com/PAS/B763).^8,9^ For samples with available RNA, targeted RNA‐sequencing was performed for rearrangement analysis in 265 genes.^9^ Sequencing of captured libraries was performed to a mean exon coverage depth of targeted regions of >500× using the Illumina HiSeq 4000 System. Sequences were analyzed for genomic alterations, including short variant alterations (base substitutions, insertions, and deletions), copy number alterations (focal amplifications and homozygous deletions), and select gene fusions or rearrangements.^8,10,11^ To maximize mutation detection accuracy (sensitivity and specificity) in impure clinical specimens, our CGP had previously been optimized and validated to detect base substitutions at a ≥5% mutant allele frequency, indels with a ≥10% mutant allele frequency with ≥99% accuracy, and fusions occurring within baited introns/exons with >99% sensitivity.^8^ Germline versus somatic status of pathogenic alterations was not delineated. Copy number analysis to detect homozygous deletions and gene-level amplifications at tetraploidy or greater was performed as previously described.^8^ TMB was determined on 0.8 to 1.1 Mbp of sequenced DNA.^11^ Microsatellite instability was determined on up to 114 loci.^12^

### Analyses of Sarcoma Subtypes With Recurrent *AXL* Mutations

For the series of sarcoma cases sharing recurrent *AXL* mutations in a similar tumor subtype, we extracted clinicopathological data including patient age, sex, and tumor site from the accompanying pathology reports. Primary site data were not available for a subset of cases. Histopathology was reassessed on routine hematoxylin and eosin stain–stained slides of tissue sections submitted for genomic profiling by 2 board-certified pathologists (E.A.W. and E.A.M.).^13^

Differences among categorical variables were assessed using the Fisher exact test or the χ^2^ test with Yates continuity correction, depending on the number of patient cases. For comparisons of age and TMB between two groups, the nonparametric Mann-Whitney *U* test was used, to avoid assumptions of normality. The Bonferroni correction was applied for multiple comparisons, and a corrected 2‐tailed *P* value of <0.05 was considered statistically significant.

### Review of Publicly Available Data Set

The Cancer Genome Atlas (TCGA) Network’s sarcoma genomic data set^4^ was interrogated for sarcomas carrying any recurrent *AXL* variant in a specific sarcoma lineage that we found in our own database. Histopathology of the single TCGA case identified in this manner was reviewed by 2 board-certified pathologists (E.A.W. and E.A.M.). Levels of *AXL* mRNA expression in this case and in the TCGA sarcoma data set overall were also examined.

### AXL Complementary DNA Expression Vector Construction and Site-directed Mutagenesis

To assess possible functional effects from a recurrent *AXL* gene mutation, we obtained the pLVX-TetOne-Puro-hAXL expression vector for wildtype (WT) AXL protein as a gift from Kenneth Pienta (Addgene Plasmid # 124797; http://n2t.net/addgene:124797; RRID:Addgene_124797). The *AXL* W451C mutation that we recurrently observed in human MFS samples (“Results”) was engineered into the pLVX-TetOne-Puro-hAXL construct by site-directed mutagenesis using the QuikChange II XL kit (Agilent) as directed by the manufacturer, to generate the pLVX-TetOne-Puro-hAXL(c.G1353C, p.W451C) expression vector. Primer sequences used for the mutagenesis reactions were as follows, with the mutated nucleotide underlined and the mutated sequence in the expression vector subsequently verified by Sanger sequencing:

AXL p.W451C Fwd: 5′-gccttctcgtggccctggtgctatgtactgctaggagcag-3′

AXL p.W451C Rev: 5′-ctgctcctagcagtacatagcaccagggccacgagaaggc-3′

### Lentiviral Production and Infection

The pLVX-TetOne-Puro-hAXL WT and the mutant hAXL(c.G1353C, p.W451C) expression vectors were separately cotransfected into HEK-293T cells with the lentiviral packaging carrier plasmid pLP VSV-G (Nova Lifetech, Cat# PVT2326) and the lentiviral packaging plasmid psPAX2 (a gift from Didier Trono, Addgene plasmid # 12260; http://n2t.net/addgene:12260; RRID:Addgene_12260) using *Trans*IT-Lenti Transfection Reagent (Mirus, Cat# MIR 6604) following the manufacturer’s recommended protocol. Virus-containing medium was harvested 48 hours after transfection, purified by passage through a 0.45 μm pore-size filter, and used to transduce recipient HEK-293T cells in the presence of 8 μg polybrene/mL (Sigma-Aldrich, Cat# TR-1003). The transduced cells were selected by 2 μg puromycin/mL. Induction of expression of WT and W451C-mutant AXL protein was achieved with 0.1 μg doxycycline/mL, applied 96 hours before harvesting cellular protein for further analyses. Negative control HEK-293T cells were transduced and selected, but received buffer instead of doxycycline.

### Immunoblots

Total protein was extracted from transduced HEK-293T cells by adding lysis buffer (1% Triton X-100, 0.5% NP-40, 150 mM NaCl, 50 mM Tris-HCl, pH 7.4, 10% glycerol) supplemented with inhibitors of proteases (Roche, Cat# 11836170001) and phosphatases (Roche, Cat# 4906837001). Protein concentration was determined by using the Detergent-Compatible Protein Assay (Bio-Rad, Cat# 500-0116). Cellular proteins were resolved by Sodium dodecyl-sulfate polyacrylamide gel electrophoresis (SDS-PAGE) and then immunoblotted following standard biochemical techniques, under nonreducing (6X sample buffer: 375 mM Tris-HCl, pH 6.8, 9% SDS, 0.03% bromophenol blue, 50% glycerol) and reducing (4X sample buffer: 200 mM Tris-HCl, pH 6.8, 8% SDS, 0.4% bromophenol blue, 40% glycerol, 5% β-mercaptoethanol) conditions.

Primary antibodies detected phospho-AXL at Tyr779 (pY779-AXL, Invitrogen, Cat# MA5-24334), total AXL (t-AXL, Cell Signaling, Cat# 8661), phospho-ERK1/2 at Tyr204 (pY204-ERK, Cell Signaling, Cat# 4377S),^14^ total ERK1/2 (t-ERK, Cell Signaling, Cat# 9102), α-tubulin (Santa Cruz Biotechnology, Cat# SC-23948), and β-actin (Sigma-Aldrich, Cat# A5441). The last 2 targets were used as loading controls. All primary antibodies were used at 1:1000 dilutions, except antibodies to detect the loading controls, which were used at 1:5000 dilutions. The signal was developed by using WesternBright™ ECL (Cat#K-12045) from Advansta, USA. The band intensity was quantified by ImageJ 1.54d software. The significance in the protein level was calculated by Prism 9 using unpaired *t*-test analysis.

## RESULTS

The single nucleotide variant (SNV) status of the *AXL* gene (NM_021913) was reviewed in each of the 19,879 unique sarcomas in our own database. Two hundred thirty-six SNVs of *AXL* were identified in 228 sarcomas, listed in Supplemental Table 2 (Supplemental Digital Content 2, http://links.lww.com/PAS/B764). Only 29 of these variants, however, were recurrent (displayed in Supplemental Table 3, Supplemental Digital Content 3, http://links.lww.com/PAS/B765). The sole *AXL* mutations for which there were multiple cases of the same sarcoma subtype were W451C and W450C. Each of the remaining 27 recurrent SNVs that we identified did not show recurrence in a single sarcoma subtype (Supplemental Table 3, Supplemental Digital Content 3, http://links.lww.com/PAS/B765). Detailed clinicopathologic and genomic features of the 8 *AXL* W451C-mutant and 2 *AXL* W450C-mutant sarcomas are shown in Table 1. A single patient case had both primary tumor and lymph node metastasis sequenced sites (case #3), each of which harbored an *AXL* W451C mutation. Of note, the variant allele frequency for the *AXL* alteration in the 11 samples was high (median: 31%, Supplemental Table 2, Supplemental Digital Content 2, http://links.lww.com/PAS/B764). No *AXL* W451C mutations were identified in any of the 443,667 non-sarcoma tumors.

**TABLE 1:** Detailed Clinicopathologic and Molecular Features of All 9 *AXL* W451C-mutant (cases #1-9) and 2 *AXL* W450C-mutant (Cases #10, 11) Sarcoma

Patient case	Age (y)/sex	Primary tumor site	Anatomic location of the sequenced tumor	Primary tumor size (cm)	Diagnosis	Grade (FNCLCC)	Mitoses/10 HPF	Necrosis	Immunohistochemistry	TMB (mut/Mb)	Genomic alterations (in addition to *AXL* mutations)	Disease and/or survival status
1	74/M	Left thigh	Left thigh	At least 14.9	MFS	3	8	Present (<50%)	CD34: focally positive S100: negative Additional IHC performed on adrenal metastasis: vimentin-positive, TP53 >50% positive, AE1/3, S100, and p16 negative	1.6	*TP53* p.R249G, *CDKN2A/B* and *NF2* homozygous loss	Dead of disease at 34.5 mo; adrenal metastasis
2	63/M	Unknown	Lung and pleura	CBD	MFS	2	2	Absent	None	3.8	*KRAS* p.Q61R, *CDKN2A/B* and *MTAP* homozygous loss	Dead of disease at 38 mo; lung metastasis
3	73/M	Left calf	1. Left calf 2. Left inguinal lymph node	11.3×8.5×2.0	MFS	2	8	Present (<50%)	1. Per report: CD34: focally positive Smooth muscle actin: focally positive Desmin: negative S100 protein: negative 2. Per report: negative for CD34, AE1/3, desmin, smooth muscle myosin, MART-1, S100, and SOX10	1. 6.5 2. 5.7	1. *ATM* p.E1031*, *KMT2C* p.Y800*, *CDKN2A/B* homozygous loss, *CRKL* amplification 2. *ATM* p.E1031*, *CDKN2A/B* homozygous loss	Alive with the disease at 27.5 mo; left inguinal lymph node and surrounding soft tissue metastasis
4	81/F	Back soft tissue	Left chest wall	25.0	MFS	1	2	Absent	Per report: smooth muscle actin positive, CD34 focally positive; negative for STAT6, MUC4, AE1/3, EMA, Desmin, SOX10, and S100	2.4	*ATRX* p.R1739*, *TBX3* p.R314fs*13, *CDKN2A/B* homozygous loss	Distant metastasis
5	44/M	Right thigh	Right thigh	8.0×7.0×50	UPS	3	>40	Present (<50%)	Per report: vimentin and CD68 positive; negative for S100, desmin, smooth muscle actin, and muscle-specific actin	4.0	*RB1* p.R251fs*11	Not available
6	80/M	Sternocleidomastoid muscle	Sternocleidomastoid muscle recurrence	6.5×4.0×3.0	MFS	2	3	Present (<50%)	Per report: EMA positive in isolated cells; negative for AE1/3, smooth muscle actin, desmin, caldesmon, and S100	4.1	*TP53* p.L330P, *CREBBP* p.Q2203fs*99, *SDHB* p.A6fs*56, *CDKN2A/B* and *PTEN* homozygous loss, *CDK6* amplification	Not available
7	70/M	Left lower extremity soft tissue	Lung	CBD	Low-grade sarcoma	1	3	Absent	Per report: negative for S100, actin, STAT6, and ALK	3.3	*CDKN2A/B* homozygous loss, *BIRC3* and *RAD21* amplification	Dead of disease at 4 mo; lung metastasis
8	78/M	Left thigh	Left thigh	CBD	UPS	2	8	Absent	None	4.9	*KRAS* p.V14I, *CDKN2A/B* homozygous loss, *BIRC3* amplification	Not available
9*	52/M	Unknown	Retroperitoneum	27.5	UPS	3	11	Present (<50%)	None	Not available	*PIK3CA* p.Q546K	Dead of disease at 20 mo
10	48/F	Left axillary soft tissue	Left axillary soft tissue	CBD	MFS	2	4	Present (<50%)	None	4.1	*RB1* p.N258fs*11, *PIK3CA* p.H1047I, *CDKN2A* homozygous loss	Lung metastasis
11	68/M	Unknown	Lung	CBD	UPS	3	12	Present (<50%)	Per report: negative for AE1/3, SOX10, S100, and p40	2.5	*SGK1* Y263*	Dead of disease at 24 mo; lung metastasis

* TCGA case.

CBD indicates cannot be determined; FNCLCC, Fédération Nationale des Centres de Lutte Contre le Cancer (National Federation of Centers for the Fight Against Cancer); IHC, immunohistochemistry; HPF, high power field.

**FIGURE 1:**
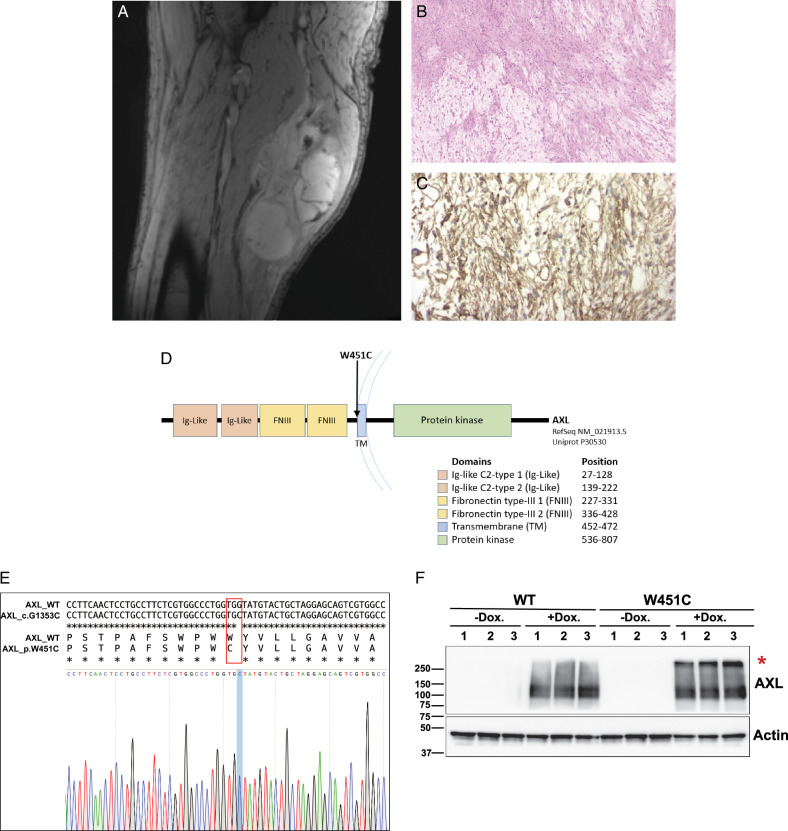
Index patient with *AXL* W451C-mutant MFS. A, T2-weighted MRI at initial presentation of the left thigh demonstrates a large multiseptated mass with internal T2 hyperintensity suggestive of myxoid components (case #1). Histopathologic examination showed prominent myxoid material (B); hematoxylin and eosin stain) and there is a focal strong expression of CD34 on immunohistochemical staining (C). On genomic profiling, the MFS harbored an *AXL* W451C mutation, corresponding to the base of the extracellular domain of the protein product, as depicted in the diagram, in which the blue arc represents the plasma membrane (D). Sequencing also showed a *TP53* p.R249G mutation and homozygous loss of *CDKN2A/B*. An *AXL* W451C expression vector was developed and verified by Sanger sequencing (E). HEK-293T cells were transduced with either WT *AXL* cDNA or *AXL* W451C, and a nonreducing Western blot of protein lysates extracted from these cells revealed that *AXL* W451C, and not AXL-WT, resulted in dimerization (red asterisk; (F). cDNA indicates complementary DNA; MRI, magnetic resonance imaging; TM, transmembrane.

Clinical imaging, histopathology, and immunohistochemistry of case #1 are shown (Fig. 1A–C), along with a diagram of the protein structure of AXL and the W451C mutation site, which is located at the base of the extracellular domain of the protein product (Fig. 1D). Characteristic histopathologic features of additional *AXL* W451C-mutant cases are showed in Supplemental Figure 1 (Supplemental Digital Content 4, http://links.lww.com/PAS/B766).

The *AXL* W451C-mutant and W450C-mutant cases showed similar clinical features and similar concurrent molecular alterations as in *AXL*-WT MFS, which we defined as MFS without *AXL* W451C or W450C mutations and without *AXL* gene amplification (Table 2).

**TABLE 2:** Comparative Demographics and Percentage Frequency of Genomic Alterations Stratified by *AXL* Status, With *P* Values.

	*AXL* W451C and W450C sarcoma (SNV)	*P* (SNV vs. WT)	*AXL*-amplified MFS	*P* (amplified vs WT)	*AXL*-WT MFS
No. cases	10	—	8	—	301
% male	80 (8/10)	0.33	75 (6/8)	0.48	60 (180/301)
Median age (y); range	72 (44-81)	0.42	72 (21-84)	0.27	66 (16-89+)
TMB (mut/Mb); Q1-Q3	3.9 (2.5-4.1)	0.0051	3.2 (2.0-4.4)	0.12	2.4 (1.6-3.5)
% MSI high	0 (0/10)	1	13 (1/8)	0.051	0.3 (1/301)
*TP53*	20 (2/10)	0.023	63 (5/8)	1	58 (174/301)
*CDKN2A*	80 (8/10)	0.0026	50 (4/8)	0.27	31 (94/301)
*RB1*	20 (2/10)	0.73	0 (0/8)	0.11	29 (88/301)
*ATRX*	10 (1/10)	0.70	0 (0/8)	0.36	19 (56/301)
*NF1*	0 (0/10)	0.22	25 (2/8)	0.64	18 (55/301)
*PTEN*	10 (1/10)	0.51	0 (0/8)	1	7 (20/301)
*KRAS*	20 (2/10)	0.052	0 (0/8)	1	3 (10/301)

For percentage values, the number of positive cases over the number of evaluated cases is included in parentheses.

The Bonferroni correction for 22 simultaneous comparisons was applied, with the cutoff for significance at *P* = 0.05/22 = 0.002.

MSI indicates microsatellite instability.

To assess the role of additional *AXL* gene abnormalities in MFS, we also reviewed *AXL* copy number alterations. Sarcoma cases with gene-level amplifications at tetraploidy or greater are shown in Supplemental Table 4 (Supplemental Digital Content 5, http://links.lww.com/PAS/B767). Cases diagnosed as MFS showed more frequent *AXL* copy number gain compared with other common sarcoma categories and with sarcomas overall (Supplemental Table 5, Supplemental Digital Content 6, http://links.lww.com/PAS/B768). In particular, 2.5% of MFS showed tetraploid or greater *AXL* amplification compared with only 0.5% of non-MFS sarcoma (8/315 vs 96/19564, *P* < 0.0001, χ^2^ test with Yates correction). MFS with *AXL* amplification showed similar clinical and molecular features to *AXL*-WT MFS (Table 2). We also reviewed sarcomas with *AXL* gene rearrangements (Supplemental Table 6, Supplemental Digital Content 7, http://links.lww.com/PAS/B769), but no recurrent partners were identified and no recurrent breakpoints that preserved the AXL kinase domain were seen within any single sarcoma category.

The recurrence of the 2 *AXL* point mutations in our sarcoma data set (n = 10/19,879) prompted us to interrogate an additional database—namely, the sarcoma genomic data set of the TCGA Network. A single *AXL* W451C-mutant sarcoma was identified (one of 206 sarcomas). The case (TCGA-DX-A8BX-01A) occurred in a 52-year-old man with UPS and harbored a concurrent point mutation in *PIK3CA* (Table 1). Available data on RNA expression showed that the *AXL* mRNA level, in this case, was not conspicuously elevated, falling within the second quartile of sarcoma overall (6.48 transcripts/million; Q2 for sarcomas spans 5.8 to 6.7). Molecular analysis revealed that the tumor clustered into the MFS/UPS group across multiple platforms (mRNA expression, DNA methylation, and PARADIGM profile; data not shown).^4^

Based on our discovery of *AXL* mutations that encode a new cysteine at the base of the extracellular domain (Fig. 1D, E), we hypothesized that the novel *AXL* W451C mutation might allow the creation of an abnormal disulfide bond between pairs of mutant proteins expressing ectopic cysteine residues, resulting in unregulated dimerization of the mutant AXL protein, hence autophosphorylation and activation of downstream signaling. To test our hypothesis, we transduced HEK-293T cells with lentiviral expression vectors for either the full-length WT AXL protein or the W451C AXL mutant (verified by Sanger sequencing, Fig. 1E). Cells were treated without (negative control) or with doxycycline to activate the expression vectors.

Protein lysates extracted from these cells were evaluated by SDS-PAGE under nonreducing conditions, followed by immunoblotting with an antibody against total AXL protein (meaning phosphorylated and nonphosphorylated forms). Figure 1F shows that after induction of the W451C AXL mutant, it spontaneously dimerized, whereas the WT AXL protein did not (Fig. 1F).

**FIGURE 2:**
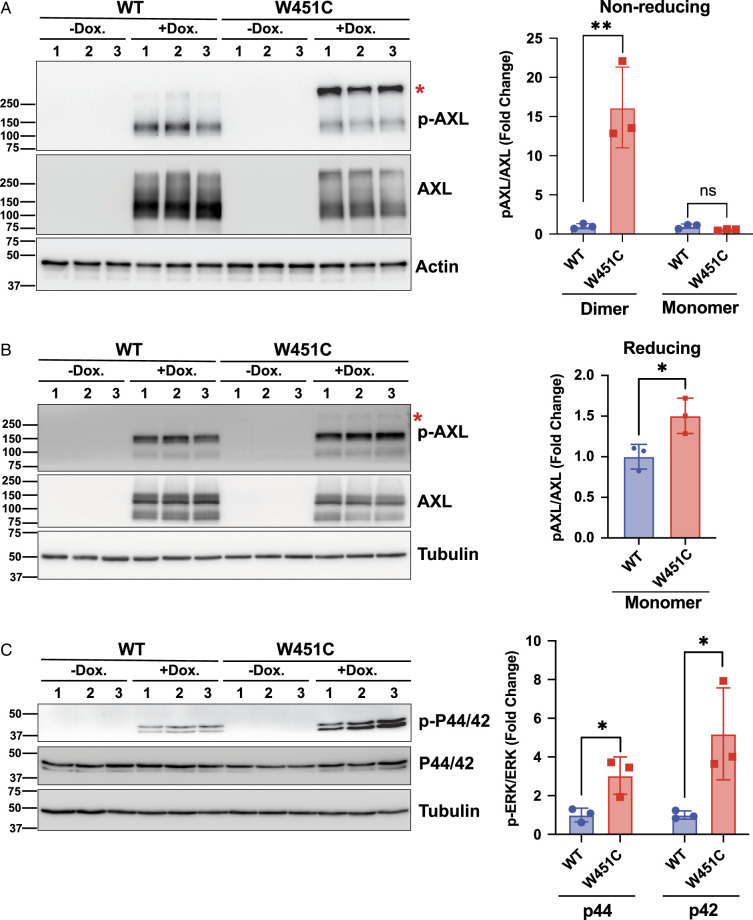
Western blots of protein lysates extracted from HEK-293T cells transduced with WT and mutant AXL isoforms. A nonreducing western blot evaluated with phospho-AXL antibody revealed phosphorylation of the dimerized form (red asterisk, (A), with a significant increase in phosphorylation as compared with WT (A). By contrast, in a reducing western blot, there was minimal evidence of dimerization (red asterisk; (B). For the monomer form seen in this reducing condition, phosphorylation of AXL is increased in *AXL* W451C as compared with AXL-WT (B). Expression of *AXL* W451C-mutant isoforms leads to increased ERK1 (p44) and ERK2 (p42) phosphorylation, as compared with AXL-WT (C) (*p<0.05, **p<0.01).

To assess functional effects, protein extracts from these cells were evaluated by SDS-PAGE under nonreducing conditions, followed by immunoblotting with an antibody specific for AXL protein that has been phosphorylated at its activation site, Tyr779 (pY779-AXL). Figure 2A shows that the dimerized form of the W451C AXL mutant was phosphorylated at this key site, whereas the small amount of higher-molecular-weight WT AXL exhibited essentially no Tyr779 phosphorylation. Monomers of neither the mutant nor the WT form showed any substantial Tyr779 phosphorylation (Fig. 2A).

In our immunoblots prepared under reducing conditions that break disulfide bonds between cysteine residues, essentially no AXL dimers remain (Fig. 2B). Accordingly, under reducing conditions, all of the pY779-AXL signal appears at the molecular weight of AXL monomers, and phosphorylation of AXL is still increased in the AXL W451C-mutant over AXL-WT (Fig. 2B).

To assess downstream signaling, we found that expression of the W451C AXL mutant significantly increased ERK phosphorylation at its Tyr204 site (pY204-ERK), compared with the effect from expression of the AXL-WT (Fig. 2C).

## DISCUSSION

In the current study, we sought to assess a possible role for AXL abnormalities in MFS through a search for *AXL* genomic alterations across large data sets of sarcomas. We found 2 specific novel recurrent *AXL* mutations, W451C and W450C, as well as unusually high levels of *AXL* gene amplification, as distinct genomic features of MFS. Moreover, the TCGA data set indicates high levels of *AXL* mRNA expression in MFS, even in comparison with other sarcomas.^5^ Our functional studies revealed that the *AXL* W451C mutation causes abnormal dimerization of the protein, autophosphorylation, and ligand-independent activation of downstream signaling (gain-of-function). Taken together, our findings implicate activating aberrations in *AXL* as a distinct feature of MFS and might aid in diagnosis as well as selection of available targeted therapies.

AXL is a receptor tyrosine kinase that, upon binding its ligand, growth arrest-specific gene-6, dimerizes and thereby activates key signaling pathways. Inhibitory antibody conjugates and small-molecule inhibitors have been developed that target the AXL protein and inhibit its signaling.^6,15–17^

MFS is a sarcoma with distinct histopathologic features^18^ but only nonspecific genomic findings identified previously, including typically low TMB with alterations in *TP53* and genes encoding proteins in the cell cycle.^1,2^ Prior methylation array studies have shown that MFS and most UPS cluster together, supporting that they represent a histopathologic spectrum of a single disease.^4^ MFS shows the highest *AXL* mRNA expression level in the TCGA data set compared with normal tissues, other cancer types, and even other sarcoma types.^5^ Of note, the single *AXL* W451C-mutant case in the TCGA did not show markedly increased *AXL* mRNA levels.^4^ This finding is consistent with our functional studies showing that the W451C mutation enhances AXL signaling even at relatively unremarkable absolute levels of expression.

Amongst *AXL-*WT cases of MFS, which we defined as MFS without *AXL* W451C or W450C mutations and without *AXL* gene amplification, we confirmed prior work on the TCGA data set indicating high levels of *AXL* mRNA.^5^ This finding raises the possibility of critical epigenetic processes in *AXL*-WT MFS that upregulate AXL expression or signaling. A potentially analogous process is the modulation of MED12 in leiomyosarcoma, in which *MED12* point mutations are rare events that disrupt MED12 function, while the majority of leiomyosarcoma show epigenetic downregulation of MED12.^19^ In MFS, the *AXL* W451C point mutation is a rare, but specific, alteration that appears to characterize a subset of the disease, while more generally, *AXL* mRNA expression is markedly elevated in a majority of MFS cases. Overall, our findings, in combination with prior work,^5,6^ suggest that AXL activation is a distinguishing and possibly defining feature of MFS/UPS and could become a specific diagnostic tool and therapeutic target. We propose that these rare tryptophan-to-cysteine mutations provide an important clue to the pathogenesis of MFS.

Both W451C and W450C alterations are tryptophan-to-cysteine, resulting in a new, potentially reactive thiol at the base of the AXL extracellular domain. Our functional studies showed that the W451C substitution allows the formation of disulfide bridges that cause ligand-independent AXL dimerization and activation. This mechanism of activation has been described for other receptor tyrosine kinases. For example, ligand-independent activation of ERBB2 has also been described as mediated by abnormal disulfide bond-induced dimerization resulting from cysteine substitutions in the juxtamembrane domain.^20^ Similarly, for DDR1, site-directed cysteine substitutions in the extracellular juxtamembrane region cause high-efficiency formation of covalent dimers, resulting in autophosphorylation.^21^

Limitations of this study include its retrospective nature and enrichment for tumors from patients with aggressive disease, including many neoplasms metastatic to distant sites. The submission of specimens for sequencing was presumably driven by late-stage disease. In addition, methylation array studies were performed only on the TCGA case in this study, which is particularly relevant for case #7, which was diagnosed as a low-grade sarcoma, not otherwise specified as MFS or UPS.

Additional studies are needed to identify if targeting AXL will benefit patients with MFS/UPS. A xenograft model showed that, among sarcomas, the only xenograft mice with significantly improved survival upon treatment with an inhibitory anti-AXL antibody conjugate were MFS/UPS cases.^6^ Several AXL-targeted therapeutics are in early clinical trials. Based on previous work^15,16^ and our current findings, those therapeutics could be explored with a particular focus on MFS.

Future studies could evaluate other diagnostic modalities, such as AXL testing through immunohistochemical surrogates. Regarding the AXL protein itself, we do not yet know whether the high levels of *AXL* mRNA in most MFS/UPS or high levels of *AXL* gene amplifications translate into high levels of AXL protein that could be detected clinically by immunohistochemistry. Additional studies are also needed to identify possible epigenetic events that could activate AXL expression and activity in MFS as possible novel therapeutic targets. In summary, our CGP of MFS has revealed recurrent, specific, activating alterations in *AXL* that may provide insights into MFS biology and potentially inform diagnostic and therapeutic options, including AXL-targeted agents.

## Supplementary Material

**Figure:**